# Lost in an unknown terrain: a phenomenological contribution to the understanding of existential concerns as experienced by young women in Sweden

**DOI:** 10.1080/17482631.2019.1658843

**Published:** 2019-08-27

**Authors:** Maria Lundvall, Elisabeth Lindberg, Ulrica Hörberg, Gunilla Carlsson, Lina Palmér

**Affiliations:** a Faculty of Caring Science, Work Life and Social Welfare, University of Borås, Borås, Sweden; b Department of Health and Caring Sciences, Linnaeus University, Växjö, Sweden

**Keywords:** Caring science, existential concerns, mental health, phenomenology, reflective lifeworld research, young women

## Abstract

**Purpose**: The aim of this study is to describe young women’s (16–25 years old) experiences of living with existential concerns for which they have sought support from healthcare professionals, teachers, family, or friends, among others.

**Methods**: This phenomenological study is based on a reflective lifeworld research (RLR) approach. Nine young women were interviewed about their experience of living with existential concerns.

**Results**: The results show the essential meaning of the phenomenon of “existential concerns” that can be described as living a life that is marked in a profound way by a feeling of being lost in an unknown terrain. To further understand the essential meaning, four constituents are described: the unpredictable body, longing for comprehension, playing a game, and longing to share one’s vulnerability.

**Conclusions**: Young women with existential concerns are vulnerable, as they are profoundly influenced by these concerns. They have to navigate through daily life while trying to fit in and to make their situation comprehensible. These young women have a longing to share their existential concerns with a trustworthy person, while at the same time they fear revealing their existential concerns and risking being rejected by others. A lifeworld-led, caring science approach, intertwined with the results of the present study, has the potential to direct caring practice.

## Introduction

This study focuses on young women^1^ in Sweden living a life with existential concerns for which they have sought support from healthcare professionals, teachers, family, or friends, among others. According to Hiltunen (2017), young adults feel that they have good physical health and are generally very satisfied with their lives. At the same time, a high percentage of young adults report that they feel inner stress, fatigue, and depression, and have difficulties falling asleep. In Sweden, there has been an increase in mental health issues such as worry, anxiety, and feeling down (Folkhälsomyndigheten, 2016; Lager, Berlin, Heimerson, & Danielsson, 2012). Approximately 42% of young women report feeling tense, anxious, and nervous (Folkhälsomyndigheten, 2016), while 35% of 15-year-old girls report they have felt low more than once a week over the past six months (Bremberg & Dalman, 2015). These issues are not unique to Sweden and are also seen in other European countries (Barry, Kuosmanen, & Clarke, 2017; Van Droogenbroeck, Spruyt, & Keppens, 2018). Feelings of worries and anxiety can cause mental health problems, but at the same time, they are a natural part of human existence. As described by Yalom (1980), existential concerns are always present and a natural part of human life, as they embrace meanings of death, freedom, dependence, vulnerability, loneliness, lostness, and homelessness.

Existential considerations may be triggered during the development from childhood to young adulthood or as part of the ageing process (Yalom, 1980). In a young woman, existential concerns may occur when questions about identity arise and as she grows into the adult world. One question that often arises concerns how to live a meaningful life. This question is often present in life, but it becomes more visible to young women when taking on new roles as they grow into adulthood (Hwang, Frisén, & Nilsson, 2018). Existing as a human being means living with the challenges of life and finding a meaning to life when performing daily tasks. Young women have their whole future ahead of them—a future that ought to involve possibilities and the freedom to choose how to live out their lives (Bhy, 2009). For some, such freedom may mean that choices feel too difficult to make and there is a risk of getting lost in terms of society’s normative view of a good life and one’s personal expectations of an improved life (Åsbring & Hochwälder, 2009; Eckersley, 2011). Existential dimensions therefore need to be addressed for young adults to feel well and handle life situations (Jacobsen, 2007). For young women (in their study, 13 to 19 years old), Larsson, Sundler, and Ekebergh (2013) emphasize the importance of relationships with others for their well-being and health. To be somebody for someone else is necessary to feel anchored in life. It may be crucial to address young adults’ mental health issues as early as possible to ensure that they receive support in managing their situation and to grow as individuals, with positive health effects for the future (McCloughen, Foster, Huws‐Thomas, & Delgado, 2012; Williams, Holmbeck, & Greenley, 2002). Strömbäck (2014) highlights the need for young women who feel mentally unhealthy to talk about their existential concerns in order to feel well.

From a caring science perspective, the purpose of care, according to Dahlberg, Todres, and Galvin (2009), is to strengthen patients’ health processes to achieve health. Health includes the whole human being and is a sense of balance and equilibrium in relation to life and the people around oneself. In the field of healthcare, patient statements are often interpreted within a medical framework, such as mental illness. Although valid, this is, of course, not the only framework. In order to recognize the complexity of health, we also need to understand health in relation to our existence (Dahlberg et al., 2009; Todres, Galvin, & Dahlberg, 2014). A recent study focused on healthcare professionals’ lived experiences of conversations with young adults with existential concerns. The study made it clear that healthcare professionals are themselves affected when young adults express their existential concerns, and they need support to strengthen their ability to stay present and help create a welcoming atmosphere (Lundvall, Lindberg, Hörberg, Palmér, & Carlsson, 2018). In order to develop caring practices that enable young women to feel well and to handle their existential concerns in a supportive manner, it is clearly important to research young women’s experiences of living with existential concerns. Thus, the present study aims to focus on existential concerns as a phenomenon that is lived by young women and which is not defined as purely mental or physical.

### Aim

The study aims to describe young women’s lived experiences of existential concerns for which they have sought support from healthcare professionals, teachers, family, or friends, among others.

### Approach and method

This phenomenological study is based on a reflective lifeworld research (RLR) approach (Dahlberg, Dahlberg, & Nyström, 2008). RLR finds its epistemological basis in phenomenological philosophy, mainly as described by Husserl and Merleau-Ponty. RLR is phenomenon-oriented and characterized by the fact that it is the phenomenon that is in focus, during the whole research process, both in data collection and in the analysis. In the present study, the phenomenon is *existential concerns* as they are experienced by young women who have sought support in the healthcare system as well as from family members, close friends, or the school environment, among others. Some important epistemological ideas in the phenomenological philosophical tradition, elaborated on by Dahlberg et al. (2008), are that human beings are immersed in the natural attitude in which aspects of everyday life are unreflective and taken for granted as well as that human consciousness is intentional. Intentionality means that human consciousness is always directed towards something other than itself; the world is perceived as something through our consciousness and given meaning (Dahlberg et al., 2008). In order to grasp a phenomenon, a scientific attitude—in this case, a phenomenological attitude—is required. This means being aware of the natural attitude and intentionality in order to approach the phenomenon in question in a scientific way (Dahlberg et al., 2008). This ensures that the researcher does not attempt to make definite what is, in fact, indefinite (Dahlberg & Dahlberg, 2003). In order to maintain a phenomenological attitude, the methodological principles in RLR refer to the openness, flexibility, and bridling of the process of understanding throughout the research. Openness is achieved through bridling with a reflective attitude and maintaining flexibility regarding the phenomenon as it appears in the data. Bridling succeeds by constantly asking reflective and critical questions of both the data and oneself—for example, “Why do I understand it like this—could it mean something else?” (Dahlberg et al., 2008).

### Participants

To describe the lived experience of existential concerns by young women, lifeworld interviews were conducted with nine young women according to RLR principles (Dahlberg et al., 2008). The inclusion criteria were that the young women were between 16 and 25 years of age and had lived experience of existential concerns for which that they had sought support. The places or people from whom they had sought support were part of the healthcare system, school healthcare facilities, family members, or close friends, among others. The included women’s ages varied between 17 and 25 years, and they came from either a big city or a rural area in the west of Sweden. Four of the young women were students in high school or university, and five were working. They were all native Swedes. The informants were contacted in one of two ways. In the first, healthcare professionals from different centres where the young women had sought support asked prospective participants if they were interested in getting more information about the study. If the participant wanted to learn more about the study, the caregiver gave the participant’s phone number to the researcher (ML). ML then contacted the participant to give more information about the study, and if the young woman wanted to participate, an interview took place. The second way was through advertising by posters in various centres where young women might seek support. In this case, a young woman could contact ML for more information about the study, and if she wanted to participate, an interview took place.

### Data collection

In order to describe the lived experience of the phenomenon as experienced by young women, lifeworld interviews were conducted in accordance with RLR (Dahlberg et al., 2008). The individual interviews were audiotaped, and the phenomenon was explored with an open and reflective attitude. Each interview began with the participant being invited to talk about the phenomenon in an open way. The phenomenon was further explored during the interview through follow-up questions. Examples include the following: “Can you explain more?,” “How did that make you feel?,” and “Can you give examples of … ?” Nine interviews were conducted. One interview took place in the home environment, and the rest took place at the interviewer’s workplace, as per the participants’ own wishes. The interviews lasted between 43 and 74 minutes, with an average length of 60 minutes. All the interviews were transcribed verbatim.

### Data analysis

The phenomenological analysis was conducted according to the methodological principles of RLR, which are openness, flexibility, and bridling (Dahlberg et al., 2008). During the process of analysis, the researcher strives to maintain an open mind towards the phenomenon as well as a bridling of their natural attitude, so they do not proceed too quickly to describe the “otherness” of the phenomenon and not just their own understanding of it (Dahlberg et al., 2008). In order to stimulate the participants to reflect on their experience, the interviewer asked questions that were open and flexible towards the phenomenon. The entire analytical process was an endeavour to describe the phenomenon’s essential meaning structure. This involves describing variations of meaning and patterns in order to identify the phenomenon’s structure of essential meanings. This process represents a movement between the whole and the parts, leading to a new whole—in this case, a movement between the interviews and particular meanings, which are lived contextual nuances, towards essential, abstract, or general meanings. The analysis can also be described as going back and forth between the whole (interviews) and the parts (meanings in the data) to describe the new whole (essential meaning) (Dahlberg et al., 2008).

First, familiarization with the data was needed. This was achieved by reading the interview transcripts several times to create an understanding of the whole text. The analysis then proceeded with a search for meaning units in the text, marked with a word or sentence related to the phenomenon. The meaning units were then clustered together. The clusters combined meanings that in some way seemed to belong together in relation to the phenomenon. This was a first step in structuring meaning in the search for patterns. In order not to diminish any nuances or make hasty conclusions, reflective questions were asked of the text, such as “Why do I understand that in this way?,” “Could that mean something else?,” or “How does it appear in the text?” The use of reflective questions in the analysis of the whole and the parts helped to counter any risk of diminishing or misunderstanding the data. This part of the analysis proceeded until all the identified meanings were clustered. Clusters are groups of meanings that seem, in some way or another, to belong together. Once the clusters were established, the essential meaning of the phenomenon began to appear. The essential meaning thus became visible through all the clusters and was the unique element that described the phenomenon of existential concern as experienced by young women. In order to further describe the essential meaning of the phenomenon, elements of meaning (constituents) were written down. Constituents are variations of the essential meaning at a more contextual and concrete level.

### Ethical considerations

This study was conducted in accordance with the research principles described in the Helsinki Declaration (World Medical Association, 2013) and follows Swedish research ethics guidelines (Utbildningsdepartementet, SFS 2003:460). Ethical approval was sought and received from the Ethics Examination Board in Gothenburg (Dnr: 483-16), and a supplementary application was approved for approaching young adult informants (Dnr: T322-18). Young women with existential concerns are an exposed and vulnerable group of people, and interviews may reveal things that the young women have not previously told anyone. This may stir up feelings of concern. Here, it was important that the young adults were fully aware of what volunteering entailed and knew they had the right to interrupt or cease participating at any time. All participants were given both written and verbal information about the study’s aim, and confidentiality was emphasized. If a young woman needed to talk further about her situation after the interview, the researcher, who is a public health nurse, stayed on for additional conversation or offered contact with another nurse or professional who could handle conversations with the young woman. It was very important that the researcher made time for additional conversation with the participants so that they felt comfortable before leaving the interview.

## Findings

The essential meaning of the phenomenon of existential concern can be described as living a life which is marked in a profound way by a feeling of *being lost in an unknown terrain*. For a young woman, being lost in an unknown terrain means feeling lost in life in relation to societal norms, values, and one’s own expectations, as well as having an inner wish for an ideal way of being and living. The unknown terrain thus means an uncertainty in relation to one’s body, bodily expressions, and society, expressed as norms and values. Societal and personal norms and values are intertwined, making it hard to find one’s own unique way of living and being. This leads to a young woman feeling lost in life, doubting her own value as a human being. Existential concerns become evident within these shattered borders between society and oneself. When experiencing existential concerns, a young woman is also lost in relation to her own body. This means that her body feels invaded by the existential concerns in an unknown way. The body, which had been known and secure, suddenly appears to be an unpredictable object which at any time can betray the young woman’s vulnerable situation by displaying her existential concerns for others to see. In this way, the existential concerns become embodied in a profound way, making it hard to navigate her life while being true to herself.

While striving to handle the feeling of being lost and thus trying to navigate this unknown terrain, attempts are made to control the situation by trying to escape from others and by playing a game. If the escape fails or the game ends, lostness and uncertainty are evident. In such a situation, there is an ambivalence between sharing with others and hiding one’s existential concerns. The young woman feels an intense longing to share her existential concerns with others. When sharing takes place, it creates a place to rest, which provides space and power for comprehension. The journey towards understanding oneself and one’s own life puzzle can then take place. It takes courage to show vulnerability and to reveal one’s innermost thoughts. Together with a longing to share, the fear of revealing one’s innermost thoughts, and the risk of being rejected as different and not living up to societal norms and values, is constant.

The essential meaning is conveyed by the following constituents: “the unpredictable body,” “longing for comprehension,” “playing a game,” and “longing to share one’s vulnerability.”

### The unpredictable body

Living a life as a young woman experiencing existential concerns means to live in an unpredictable body. To say the body is unpredictable means there is a tension between the known body and the way the body expresses itself in an unknown way when existential concerns are experienced. These existential concerns may manifest as physical expressions such as breathlessness, panic attacks, hearing loss, dizziness, or nausea, which reveal a compromised state of well-being. When existential concerns invade the body, it is difficult to understand what is happening and how to handle the unknown physical expressions of the body. The unpredictable body restricts everyday life and the opportunity to live life to the fullest. When unknown physical expressions appear, there is a felt need to escape from others into a safe place:

“I can even say that I should go to the toilet, just so that I can get away from people and have a moment where I get to feel bad and where it is OK, because I am myself and nobody can judge me … . Then I can also get a panic attack because all the concerns that has been built up during the day, that I have to be a certain way, puts an extreme pressure on me”.

The unpredictability of the body also means being trapped in an unknown body. In some situations, the body feels frozen, an object from which there is no way to escape in order to find relief. The feeling of bodily control is lost, which means that the body fails to live up to what is expected as normal:

“I become claustrophobic in my own body, and it happens sometimes that I wake up with my head before the body … . When you try to move and you cannot … Me and myself and my feelings are stuck in my own body, and I just want to get out, I wanted to scream”.

Having an unpredictable body means one must be constantly prepared for the body’s unpredictable physical changes, which act as a fetter in daily life. The body’s unpredictable expression thus feel difficult to control, which limits the possibility of living life as desired:

*“It is not that I have broken a leg, so I know that it will heal … .I think I will always have issues with mental illness, and it is so awful to think about that, because then I know that I will always be a little limited in what I do”*.

When a young woman experiences existential concerns the physical expressions can more easily be articulated and can be used as a protection to hide behind, which means withholding the existential concerns from others in order to prevent difficult questions. Such a shield provides protection for the young woman against others’ views and opinions that might make her feel worse. The physical expressions can then act as an explanation in order to hide the existential concerns:

“Sometimes it has been that I have to stay home from work because I do not feel good … mentally. I usually say that it is something physical—it is easier. But it doesn’t really feel good at all to lie”.

### Longing for comprehension

Living a life as a young woman experiencing existential concerns means experiencing a longing for comprehension by trying to understand oneself and the situation. The longing indicates a willingness to feel harmony with oneself. Life with existential concerns is experienced as a striving to understand how life is to be managed and how life can be meaningful for oneself*: “One does not want life to end, and so one starts to think, then one gets stressed … much of that, and what I really do with my life”.*

Time is experienced as rushing away and is a constant reminder of one’s finite existence. The existential concerns mean a fear that life will end when it has barely begun. Thoughts about the finality of life awaken an uncertainty of what to do in life, which makes life hard to understand: “*I thought all the time, every day about life, is it worth living, why should I live, why do I exist even?”*

The longing to understand and to make one’s life situation comprehensible means battling with existential concerns such as thoughts and feelings about life, its meaning, finiteness, and death: “*It may be that I am only reminded that I shall die and not, never again, live. I will be like … I stay up and feel, like, a lump in the stomach”.*

The longing for comprehension in a life with existential concerns implies a desire to have a more comfortable life situation where one is not constantly reminded of existential concerns. Trying to understand life situation, however, is exhausting and time-consuming, which appears as a tiredness that complicates everyday life: “*I barely have the energy … to go to the toilet*.” Everyday life emerges as a struggle where existential concern battles with the desire for comprehension. The struggle is also directed at determining what feels good and what gives one a sense of well-being. When the concerns are intensively present, the desire for comprehension seems so urgent that the young woman is prepared to do everything in her power to control the situation: “*I got thoughts that I wanted to hurt myself because I felt that … it might be a way out, because I feel so bad that I do not feel that I can live with this anymore”.*

### Playing a game

Living a life as a young woman experiencing existential concerns means playing a game: to play a role and live life through that role when experiencing existential concerns. At the same time, adhering to the rules of the game means trying to fit into the norms of society and not reveal one’s existential concerns to others: “*You should not and you cannot feel bad because then … you are an inferior and weaker human being than others”.*

The rules of the game are requirements and norms that society sets for a young woman, such as a pressure to be “good-looking” which involves behaving and dressing like a woman; to be clever and perform well in school and at work, which she must do in order to be given any attention and validation from others; and to do the “right” things for a young woman to do, such as being nice and quiet. When the norms do not align with one’s innermost wishes, or when the norms are opposite to one’s own will to live out life, existential concerns become evident: *“My studies mean everything to me, because I get a grade on them and it says on paper as well, that this is how good you are” “but I can’t manage to do this [get high grades] because I can’t concentrate or for some reason I can’t perform. So people just judge me as lazy”*

When a young woman is performing according to norms, she is praised and becomes someone of worth in the eyes of others and herself. Playing a game is a way of controlling one’s life without risking the loss of control, and in that way exposing one’s existential concerns and ill-being to others. Through the role she plays, a young woman tries to display well-being in order to fit in and be like others: “*I laughed a lot even though I didn’t want to and … said I felt good though I didn’t feel good—it was almost all the time, both at school and at home”.*

The role acts as a protection against the otherwise judgemental and sometimes unreasonable views of other people about how to be a young woman. There is a fear of showing one’s true self because others may see this as a weakness. It is easier to play the game of appearing to be what others expect or what one thinks is the right way, as it does not lead to questions from others. Such questions can be difficult to answer without revealing a sense of being different from what is expected: “*But sometimes after a school day I could come home and lock myself in my room and just … there I could drop the façade”.*

Playing a game also means holding up a façade during activities with others when experiencing existential concerns, which is strenuous and energy-intensive; “*it feels like you’re simply playing theatre all the time*.” To handle each day, when experiencing existential concerns, everything is carried out according to a pre-planned routine. The planning is designed to prevent the façade from being exposed:

It starts when I wake up that I have to plan exactly what to do this day. And it can’t be anything that deviates during that day. If I have decided to go to school, then I must get up, I must prepare myself, I must say good morning to all, must be nice because I am, after all, the “happy girl” in the eyes of others.

To endure and gain power to maintain the façade, attempts to withdraw to a safe place are made “*just to get away from others and have a moment to feel bad*.” To withdraw means that the game temporarily ends, but it also means being forced to refrain from activities that were previously experienced as fun. When a safe place is found to withdraw to, space is given to let out one’s true thoughts and feelings for a while: “*When I am sad and when I feel worried I am always in my room, putting on a YouTube clip and then I don’t have to do anything, just sit there by myself and do what I want”.*

Being alone is a soothing feeling. At the same time, however, loneliness means that the existential concerns are given more space and expand in a tremendous way: “*I gained easier access to my feelings, and I thought that was extremely scary*.” To withdraw from the game is therefore to experience an ambiguous feeling of both taking control and losing control. There is a desire to pause the game and be oneself for a while. At the same time, there is a fear of how to handle the emotions that may appear in front of others. In order to avoid the existential concerns taking over, these concerns are pushed away by constantly doing things that will distract:

*Just put on some movie or put on some music. I do not need to watch it, but I want it to be on so I can watch if I want, and then I’m calm for an hour and a half … I don’t have to think about anything else*.

### Longing to share one’s vulnerability

Living a life as a young woman experiencing existential concerns means longing to share one’s vulnerability. This longing to share one’s vulnerability as a young woman is strong. Succeeding means togetherness with others and not having to feel different. But sharing also means having the courage to tell others about one’s existential concerns. Having the courage to show vulnerability requires meeting with people who are open and have a willingness to understand without judging: “*I just felt like she, or that someone just for once listened to me and wanted to listen to me” “she made me feel like I was not alone in it anymore”.*

To dare to show vulnerability, there must be a trustworthy person who can act as a confidant. Showing one’s vulnerability means exposing oneself to the risk of being neglected and sometimes even rejected by the other. When courage is lacking, the existential concerns are pushed away, but they do not disappear. Instead, there is a risk that they will grow even stronger. Being neglected and rejected causes a sense of being meaningless and less worthy as a young woman. Having such an experience means that the courage to show vulnerability disappears, and there is an increasing reluctance to share: “*It is hard enough to realize that you feel bad and that you need help, and then when you stretch out a hand and get slapped in the face, then it becomes even more difficult”.*

The longing to share one’s vulnerability is intense, but at the same time, there is a fear of being a liability and a burden to the one who will receive the story. This means there is some doubt as to whether the existential concerns are worth sharing:

“One feels that one cannot always share … one does not always want to feel bad and one does not always want to be the one who, who complains or who thinks things are hard either, so sometimes it has become so natural that one has … ignored saying it”.

When the courage to show one’s vulnerability and share one’s experiences emerges in the presence of a trustworthy person, one who receives the concerns without judging or immediately suggesting standard solutions, there is a feeling of calmness. Sharing provides a place to rest and a space to catch one’s breath, and the power to find oneself in life: *“She tried to listen to me, we just talked and tried to understand my problem and the reason why I was concerned” “Even though she was in a different generation than I am, she understood me”.*

## Discussion

This study focuses on the experience of living a life with existential concerns from the perspective of young women in Sweden, a phenomenon rarely discussed in other studies. The essential meaning reveals that young women with existential concerns live a life which is profoundly influenced by these concerns. In such a life, they feel lost in an unknown terrain, striving to comprehend themselves and their lives. The results also show that the young women try to find meaning and coherence in life, striving for well-being. This struggle leads to the young woman feeling lost in life, doubting her own value as a human being. This striving for meaning is confirmed by Jacobsen (2007), who argues that the basic nature of human beings is to always seek meaning in life in order to feel well. Existential concerns can, then, be understood as a natural way of navigating and being in life and not just as an issue of mental illness that needs to be medicalised. To gain a greater understanding of the experience of living a life with existential concerns, this discussion examines the results in the light of philosophy, caring science and previous research.

The essential meaning in the present study reveals that young women live life as an unknown terrain, which means feeling uncertainty in relation to one’s body, bodily expressions, and society (expressed as norms and values). Societal and personal norms and values are intertwined, making it hard to find one’s own unique way of living and being. Young (2005) finds that young women living in a Western culture in the 2000s can be described as living in a patriarchal social structure where the ambiguity of being both a free subject and a determined object is evident. This ambiguity can be glimpsed in the results of the present study, which highlight that existential concerns become evident within the shattered borders between oneself and society. The existential concerns then awaken a vulnerability wherein a young women strives to find her place as a free subject, navigating the determined objectifying claims from her society, in which there is a need to play a game in order to fit in. It is also sometimes necessary to hide oneself in order to control one’s existential situation in having an unpredictable body. This can be further understood by relating the results to what Strömbäck, Malmgren-Olsson, and Wiklund (2013) assert about femininity, embodiment, and mental illness in a group of young women in Sweden. They highlight the need for awareness about young women’s vulnerability due to growing up in a patriarchal society. Strömbäck, Formark, Wiklund, and Malmgren-Olsson (2014) also highlight that a young woman living a stressful life experiences her body as a collapsing body, and also experiences lost access to and confidence in her own body, which can be understood as an existential loss of subjectivity and bodily presence. This seems to be in line with the present study, which puts forward that young women with existential concerns experience the body as an unpredictable object ready to betray them in their existential vulnerability.

It thus seems clear that healthcare professionals need to be aware of the vulnerability of young women seeking support for existential concerns. The vulnerability described in the present study can be understood as young women being in an exposed situation due to both the bodily (physical) and cultural claims in relation to a patriarchal society, which may influence the way they feel they should live. Here, de Beauvoir’s (1949/2002) analysis of the lived experiences of young women, as being forced by society and thus being trapped in their own bodies, seems to have value for further understanding this vulnerability. In the present study it became evident that young women feel forced by society to fit into a norm of what it means to behave as a young woman—to look and behave like a “real” woman. Thus, they feel examined and criticized by others regarding how they should look and dress, and so on, which awakens existential concerns regarding how to be a young woman and living out life. This vulnerability to existential concerns influences how young women navigate life with the ambiguity of the known and unknown body and the risk of being betrayed by their own body. In order to live life as expected by society they feel compelled to play a game and try to hide themselves.

Strömbäck (2014) identifies a need to shape a space for young women in which they can be strengthened. Such a space could be a room in which young women can spend time together and take strength from others in the same situation. This is in line with the present study wherein the young women expressed a wish to share their existential concerns with secure others. It thus seems fair to assume that there is a need for healthcare professionals to make it possible for young women to meet and share their existential thoughts with each other but also to share them with secure healthcare professionals. Healthcare grounded in caring science with a lifeworld-led perspective (Dahlberg et al., 2009) has the potential to allow the experiences of young women to be heard in order to take such experiences into account when caring for young women with existential concerns. For this to occur, both the healthcare professionals and the young women need to become aware of the vulnerable situation of women living a Western patriarchal culture. If this is done, it is fair to assume that young women may have the potential to transcend their situation, take command of their lives, and feel well.

The present study reveals that young women try to escape from others by being alone, even if they also feel a longing to share. Such sharing is in line with what Larsson et al. (2013) argue is fundamental for well-being. They mean that feeling anchored in life relationships with others is significant for young women to feel well. Thus, Larsson, Sundler, and Ekebergh (2012) also argue the importance of understanding that relationships with others can be both positive and negative for one’s well-being, depending how one is met by others. The results of the present study reveal the young women long to share their existential concerns with a trustworthy person, while at the same time they experience a fear of revealing their innermost thoughts due to the risk of being rejected by the other. This is in line with other studies that show there may be several challenges and obstacles to overcome for young adults who seek support for their well-being. Gulliver, Griffiths, and Christensen (2010) argue that young women can be ashamed of their situation and may not seek support because of the stigmatization of mental illness in society. Young women may also fear seeking support and not being taken seriously (Goodwin, Savage, & Horgan, 2016; Yap, Reavley, & Jorm, 2013), which is confirmed in the present study.

From a caring science perspective, the results of the present study show the importance of meeting young women with existential concerns in their lifeworld to create an adequate caring relationship so they can feel confident and secure enough to share their existential concerns. Hemingway (2011) suggests that in order for care to promote well-being, it is important to see the patient’s own lived experiences. To develop caring practices for young women with existential concerns, the results of the present study, together with a caring science perspective, can form a point of departure.

Concerning the young women’s sense of being lost in an unknown terrain, Galvin and Todres (2013) argue that in order to be caring, there is a need to understand the complexity of the intersubjective domain in a caring situation, expressed in the present study as a longing and a fear of sharing. The awareness of this has the potential to develop caring relationships, in which trust can develop. Such a relationship must, in order to be caring, be constituted by a shared equality. Such a caring situation can be described as both being at home and being on an adventure, thus meeting both oneself and the other in order to feel well. This is in line with Strömbäck’s (2014) suggestions regarding a shared room.

The present study reveals how important it is that care is directed towards young women as human beings with lived experiences. This means that healthcare professionals need to approach a young woman as a whole, not divided into parts such as the physical and the mental. Bullington (2013), inspired by Merleau-Ponty’s philosophy, explains this as an integrated whole, a lived body in which there are no dividing lines between body, mind, and world. The young women in the present study describe how the body is experienced as unpredictable and limits everyday life and the opportunity to fully live their lives, perhaps based on fear and a feeling that new situations that are daunting, as she faces new and unknown changes. When suffering from existential concern arises, the young woman feel lost in an unknown terrain. This way of thinking may provide an understanding of why young women feel lost in an unknown terrain, which is an obstacle to overcome.

According to Dahlberg (2011), the aim of caring, and thus of caring practice, is to support the patients’ (in this case, the young women’s) health processes. The goal of caring is health, which can be described in terms of well-being and the quality of being able to live out one’s life as desired and to manage one’s life projects. Bullington (2013) posits that care needs to bring out the experience and the story that lies behind the bodily expressions to fully understand the lived situation. This is also in line with Dahlberg’s (2019) assertion that there is a need to make room for both objective and subjective perspectives on the body, and the movement between them. Time, space, and a trusting atmosphere seem to be needed to make the approaching movement possible. It is therefore our understanding that the situation for young women with existential concerns must be understood according to both their bodily expressions and their lived experiences in order to be caring and enable the young women to live their lives while being true to themselves. Behind the objective bodily expressions, there is an experience to be shared.

### Methodological considerations

To explore and describe existential concerns using the RLR approach, based on phenomenology, it is important to keep the phenomenon in focus the whole time, from data collection to analysis and at the same time keep the objectivity (Dahlberg et al., 2008). To maintain objectivity throughout the process, van Wijngaarden, Meide, and Dahlberg (2017) suggest using openness, bridling, and compliance. It can be a methodological strain to maintain openness and a bridled attitude during the whole research process. In this study, openness was achieved through a bridled and reflected attitude, a compliance with the phenomenon, and a genuine wish to understand the phenomenon as it truly is. For example, during interviews, the interviewer tried to be open and compliant with how the phenomenon presented itself in each informant’s story by asking follow-up questions to elucidate any variations in the phenomenon’s meaning, in order to avoid drawing hasty conclusions regarding the meaning of the phenomenon. At the same time, the interviewer needed to be in close proximity to the participants to deepen their reflection on the phenomenon. Here, the interviewer had to find a balance between keeping a focus on the phenomenon and adherence to the otherness that appeared in the dialogue, which was achieved by not moving too quickly in the interview, and pausing before asking follow-up questions. To bridle the understanding throughout the analytical process, a reflective approach was taken and constantly asking critically reflective questions regarding the material and the understanding of the phenomenon was done. To ensure objectivity, the members of the research group continually discussed the findings and reflected upon them. The results were also discussed in detail in a seminar with other researchers, which contributed to the results strength and validity.

To further discuss the strength of qualitative research, van Wijngaarden et al. (2017) argue that validity is associated with meaning and generalizability comes from presenting the results as both a structure of meaning and in direct quotations from the interviews (van Wijngaarden et al., 2017). From a positivistic perspective, the low number of participants might be seen as a hindrance to generalizing the results. From a phenomenological perspective, however, the strength of the work lies not in the number of participants but in the depth of the descriptions of meanings. The variety of young women who participated in the study gave rich descriptions of the phenomenon, allowing us to describe the phenomenon in terms of its essence and constituents to deepen the understanding of the phenomenon and provide validity for the study. When it comes to generalizability, we must consider that the results are contextual, but it is reasonable to assume they are transferable to similar contexts, as the results are based on a variety of data and presented as an abstract essence.

Hopefully, the results of this study will contribute to an increased knowledge for professionals who meet young women with existential concerns in order to recognize these concerns and allow the young women to discuss them, thereby strengthening their health process.

## Conclusions and clinical implications

To conclude, the lives of young women with existential concerns are profoundly influenced by these concerns. Young women live in a patriarchal society that places special demands and requirements on them based on the norms and values of society. They must navigate their daily lives while trying to fit into these norms and values to make their situation comprehensible. Such navigation is not always achieved in terms of being true to oneself and one’s own inner wishes; instead, it may be directed to please others. Existential concerns then arise and manifest themselves in bodily expressions and feeling unwell, but behind these expressions and feelings there is a story to be told. A lifeworld-led, caring science approach, informed by the results of the present study, has the potential to direct caring practice. Such an approach is dependent on the patients’ perspective and is sensitive to the existential aspects in the young women’s stories. This, in combination with an awareness of the influence of our patriarchal society, can guide caring practices that enable young women to find a place where they are allowed to be vulnerable, in order to comprehend their existential concerns and situations.

## Note

1. In this article, we define young women and young adults as those from 16 to 25 years of age. References to other ages are given in parentheses.
